# Supplementary material for: Rational Design of a Protein Kinase A Nuclear-cytosol Translocation Reporter
Source: Sci Rep. 2020 Jun 9;10:9365. doi: 10.1038/s41598-020-66349-3 (PMC7283302; doi:10.1038/s41598-020-66349-3)
Supplement: Supplementary file 1 — Supplementary Figures 1-2. [file 41598_2020_66349_MOESM1_ESM.docx]

**Supplementary Information for:**

Rational Design of a Protein Kinase A Nuclear-cytosol Translocation Reporter

Allen K. Kim^1,2,3*^, Helen D. Wu^1,2,3^, Takanari Inoue^1,2,3*^

^1^Department of Biomedical Engineering, Johns Hopkins University, School of Medicine, Baltimore, Maryland, United States.

^2^Department of Cell Biology, Johns Hopkins University, School of Medicine, Baltimore, Maryland, United States.

^3^Center for Cell Dynamics, Johns Hopkins University, School of Medicine, Baltimore, Maryland, United States.

^*^Correspondence to [akim85@jhmi.edu](mailto:akim85@jhmi.edu), [jctinoue@jhmi.edu](mailto:jctinoue@jhmi.edu)

Content: Figures S1 and S2

Figure S1


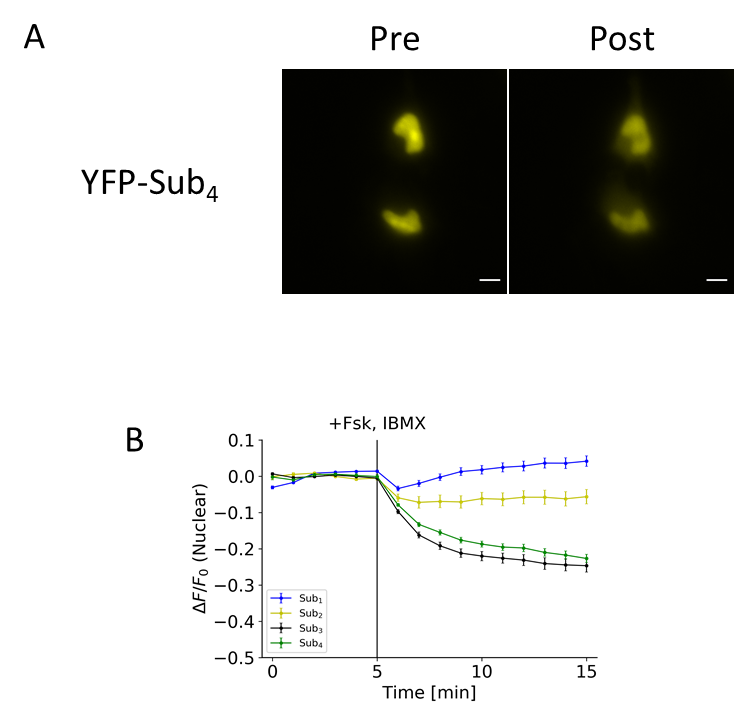


**Figure S1: Response of tetravalent substrate to PKA activation.**

1. Representative images show the localization of YFP-Sub_4_ pre-treatment (Pre) and 10 minutes post-treatment (Post). Sub_4_ peptide sequence is following: LRRASLG-K-RRASLG-K-RRASLG-K-RRASLG.
2. Response profile shows the normalized change in nuclear fluorescent signal after PKA activation with the peptides containing the different number of substrates.

All data points in this figure represent the average signal intensity calculated from 30 cells over 3 independent experiments. Error bar represents standard error of mean. Scale bar represents 10 µm.

Figure S2


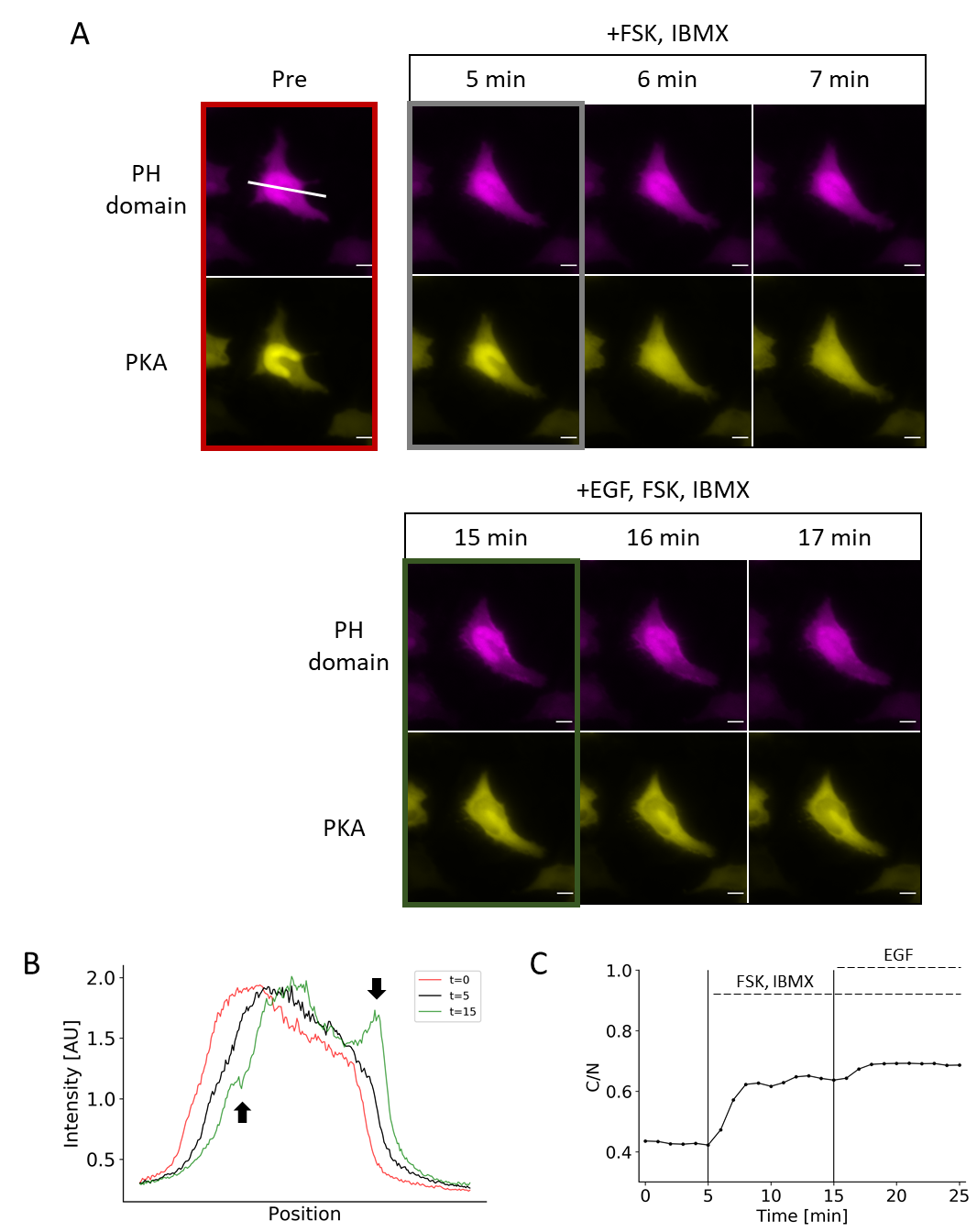


**Figure S2:** **Duplex monitoring of PIP_3_ and PKA in a single cell with PKA activation followed by EGF treatment.**

1. Representative images show PKA activity and PIP_3_ accumulation (as indicated by translocation of PH domain) in a single cell after PKA activation followed by EGF treatment.
2. Line-scan of the image in Figure 4A (white line) shows the transient appearance of enriched fluorescent signal at the plasma membrane with EGF treatment but not with PKA activation (arrows).
3. Response profile shows the normalized signal change that results from PKA activation followed by EGF stimulation.

Data points represent measurement from a single cell. Scale bar represents 10 µm.
